# Long-Term Effects of Half-Time Photodynamic Therapy on Retinal Sensitivity in Eyes with Chronic Central Serous Chorioretinopathy

**DOI:** 10.1155/2020/3190136

**Published:** 2020-08-17

**Authors:** Takehito Iwase, Hirotaka Yokouchi, Masayasu Kitahashi, Mariko Kubota-Taniai, Takayuki Baba, Shuichi Yamamoto

**Affiliations:** Department of Ophthalmology and Visual Science, Chiba University Graduate School of Medicine, Chiba, Japan

## Abstract

The purpose of this study was to evaluate the long-term effects of half-time photodynamic therapy (PDT) on the retinal sensitivity in eyes with chronic central serous chorioretinopathy (CSC). Twenty-two eyes of 22 patients with chronic CSC were studied. PDT was applied with full-dose verteporfin and half-time laser duration. The best-corrected visual acuity (BCVA) and retinal sensitivity in the central 2 and 10 degrees were evaluated at the baseline, and at 12 and 24 months after the half-time PDT. The retinal sensitivity was determined by Macular Integrity Assessment microperimetry (MAIA, Centervue, Padova, Italy). The results showed that the mean retinal sensitivities in the central 2 and 10 degrees were significantly improved at 12 months (25.6 ± 2.79 dB, median; 26.11 dB, 25.6 ± 2.25 dB, median; 25.65 dB, respectively; *P* < 0.001) and at 24 months (26.3 ± 2.62 dB, median; 27.38 dB, 26.6 ± 2.21 dB, median; 27.45 dB, respectively; *P* < 0.001) after the treatment compared to that at the baseline (19.2 ± 3.93 dB, median; 19.34 dB, 20.9 ± 2.92 dB, median; 20.9 dB, respectively). The BCVA was also significantly improved from 0.18 ± 0.19 median; 0.15 logarithm of the minimum angle of resolution (logMAR) units at the baseline to 0.07 ± 0.15 median; 0 logMAR units at 12 months (*P* < 0.001) and to 0.049 ± 0.16 median; -0.039 logMAR units at 24 months (*P* < 0.001). We conclude that half-time PDT results in a significant improvement of the mean central retinal sensitivity for at least 24 months in eyes with chronic CSC. Thus, half-time PDT is beneficial in resolving chronic CSC for a relatively long period.

## 1. Introduction

Central serous chorioretinopathy (CSC) is characterized by a serous retinal detachment in the macular area. The serous retinal detachment (SRD) is caused by a leakage of fluid from the choroid into the subretinal space by a breakdown of the barrier in the retinal pigment epithelium (RPE) [1]. Indocyanine green angiographic images have shown hyperpermeable choroidal vessels [2], and enhanced-depth imaging optical coherence tomographic (EDI-OCT) images have shown a thickened choroid in eyes with CSC [3]. Acute CSC is often a self-limiting disorder, and the visual acuity recovers after the resolution of the SRD. However, some patients have a recurrence and the eye is then defined as having chronic CSC. If left untreated, chronic CSC can progress to diffuse RPE atrophy [4].

There are several successful treatments for CSC such as focal laser photocoagulation [5, 6], and it has been recently shown that photodynamic therapy (PDT) with verteporfin is a very effective treatment [7, 8]. Hyperperfusion and hyperpermeability of the choriocapillaris appear to be the primary alterations in the early stage of acute and chronic CSC. Laser photocoagulation can stop the leakage of fluid from the RPE by coagulating the leakage points, but it is not specific enough in resolving the fundamental choroidal vascular problem [9].

It is believed that the mechanism for resolving the SRD by PDT with verteporfin might be that the PDT induces vascular endothelial damage and thrombus formation due to a short-term choriocapillaris occlusion and a long-term choroidal vascular remodeling. These alterations lead to a normalization of the dilated choroidal vessels and a reduction in choroidal congestion, vascular hyperpermeability, and extravascular leakage. As a result, the SRD would improve in the eyes with CSC [7–9].

PDT treatment for chronic CSC has been reported to result in positive visual outcomes in most studies [7–9]. However, it has been reported that conventional PDT occasionally had serious side-effects such as RPE atrophy, choroidal ischemia, and secondary choroidal neovascularization [7, 9]. To mitigate these adverse side effects, several studies have reported using a safety-enhanced PDT with one-half of the conventional dose of verteporfin (3 mg/m^2^) [10–12] or with reduced fluence of 25 J/cm^2^ for 83 seconds, i.e., 300 mW/cm^2^, in eyes with chronic CSC [13, 14].

In addition, it has been reported that PDT with half the usual exposure time was reported to be a helpful treatment for chronic CSC [15, 16]. These modified PDT applications had sufficient photodynamic effects on chronic CSC with fewer complications and side effects compared to conventional PDT.

Although visual acuity is an important parameter of the overall visual function, it tests only the foveal function, and some patients with chronic CSC with good visual acuity might have poor contrast sensitivity, color discrimination, dark-adaptation, and macular sensitivity [17–20]. Some studies have shown that patients with resolved CSC and good visual acuity have significantly lower central retinal sensitivity [21, 22]. Thus, the assessment of the BCVA alone may give false information of the overall visual functioning of an individual.

The Macular Integrity Assessment Microperimeter (MAIA, Centervue, Padova, Italy) is a relatively new instrument that can obtain digital fundus images with automated microperimetry. Improvements of the retinal sensitivity around the macular before and after half-dose PDT, [12, 23] half-fluence PDT, [14, 24], and half-time PDT [16] have been recently reported for eyes with chronic CSC. Senturk et al. reported that the retinal sensitivity was improved 6 months after half-dose PDT [12], and Fujita et al. similarly reported that the retinal sensitivity was improved 12 months after half-dose PDT [23]. Casalino et al. reported that retinal sensitivity was improved 2 months after half-fluence PDT [24], and Reibaldi et al. reported that retinal sensitivity was improved 12 months after half-fluence PDT [14]. Muslubas et al. reported that retinal sensitivity was improved 3 months after half-time PDT [16]. These findings indicated that PDT can have good results for at least 12 months. Although Sheptulin et al. used half-time PDT on a large cohort of chronic CSC eyes, they evaluated the retinal function only by the BCVA [15]. To the best of our knowledge, there have not been any studies that determined the changes of retinal sensitivity and BCVA at 24 months after half-time PDT.

Thus, the purpose of this study was to determine the effects of half-time PDT on the macular sensitivity and BCVA after 24 months in eyes with chronic CSC.

## 2. Materials and Methods

### 2.1. Study Participants and Statement of Ethics

This was a retrospective, observational case series of 22 eyes (right 10 and left 12) from 22 patients with chronic CSC of ≥6 months duration. The procedures used in this study were approved by the Institutional Review Board of Chiba University Graduate School of Medicine (No.2601). A signed written informed consent was obtained from all patients. The procedures of this study followed the tenets of the Declaration of Helsinki.

The inclusion criteria were the presence of SRD involving the fovea in the optical coherence tomographic (OCT) images of at least 6 months duration. All patients had the following characteristics: axial length between 21.0 mm and 26.5 mm or refractive error between -6 and +6 diopters; intraocular pressure between 10 to 20 mmHg; and no uveitis and glaucoma. Patients who had evidence of choroidal neovascularization, polypoidal choroidal vasculopathy, or other maculopathies documented by fluorescein angiography (FA) or indocyanine green angiography (ICGA) were excluded. Patients who had undergone PDT two or more times were also excluded when they had a recurrence of a SRD.

A chronic CSC refers to a prolonged absence of a spontaneous resolution of the SRD involving the macula and is associated with the idiopathic leakage from the RPE during fluorescein angiography. ICGA was used to confirm the presence of choroidal vascular hyperpermeability, to determine the size of the hyperpermeability to guide the PDT if indicated, and to exclude the presence of polypoidal choroidal vasculopathy or choroidal neovascularization. A history of the medical and ocular disease was obtained from the medical records of each patient, and a complete ophthalmic examination including determination of the best-corrected visual acuity (BCVA), slit-lamp biomicroscopy, OCT, FA, and ICGA was performed. All OCT images were obtained with the Heidelberg Spectralis OCT instrument (Heidelberg Engineering, Heidelberg, Germany) at the baseline, and at 12 and 24 months after the treatment. The measurements from the outer border of retinal pigment epithelium (RPE) to the inner border of the sclera were used to determine the subfoveal choroidal thickness (CT) by using the enhanced depth imaging (EDI) algorithm.

### 2.2. Modified PDT Protocol for Chronic CSC

A modified PDT protocol for chronic CSC was used. The patients received an intravenous injection of verteporfin (Visudyne; Novartis Pharma, Tokyo, Japan) of 6 mg/m^2^ body surface area. Fifteen minutes later, the eye was irradiated by a diode laser (689 nm) for 42 seconds with a fluence of 50 J/cm^2^ using the Visulus PDT system 690S (Carl Zeiss Mediac AG, Jene, Germany). The exposure time was set at one-half of the standard setting to reduce the total energy to one-half of that of standard fluence-PDT. The area of the laser spot was determined by the size of the lesion in the ICGA images, and the size covered the entire choroidal vascular hyperpermeability lesion plus a margin of 1000 *μ*m. All PDT treatments were performed by the same medical retina specialist (HY). After the treatment, the patients were instructed to wear protective spectacles and to avoid strong light for 48 h.

### 2.3. Macular Integrity Assessment Microperimetry (MAIA)

An assessment of the location and stability of the fixation was determined by Macular Integrity Assessment microperimetry (MAIA, Centervue, Padova, Italy). The threshold macular sensitivity and fixation stability were determined with the MAIA device. MAIA is a nonmydriatic, near-infrared, line-scanning, laser ophthalmoscope, which incorporates a high-frequency eye tracker and an automated threshold fundus perimeter. The automated eye tracker locks onto the entire fundus image and records fixation changes 25 times/sec during the testing. The software identifies the preferred retinal locus that identifies the patient's preferred fixation site. An automated retinal sensitivity program using a Goldmann III stimuli size on a grid of 37 stimuli covering an area of 10 degrees centered on the patient's preferred retinal location.

### 2.4. Statistical Analyses

Statistical analyses were performed with the SPSS software for Microsoft Windows (SPSS V.26, IBM Japan, Tokyo). Wilcoxon signed-rank tests were used to determine whether retinal sensitivity, the BCVA, and the CT at each posttreatment time were significantly different from that at baseline. Spearman's rank correlation test was performed to determine the significance of the correlations between the BCVA and retinal sensitivity at the baseline, 12 and 24 months after treatment and between the changes in the BCVA (logMAR units) and the retinal sensitivity from the baseline to 24 months after the treatment. A *P* < 0.05 was taken to be statistically significant.

## 3. Results

All 22 patients were Japanese (18 men, 4 women), and their mean age was 57.5 ± 11.0 years with a range of 32 to 73 years. The demographics of the patients are shown in Table 1.

All eyes had typical signs of CSC at the baseline with fluorescein leakage, dilated choroidal vasculature with hyperpermeability, late extravascular leakage at the macula in the ICGA images, and the SRD in the OCT images. None of the patients had a new leakage in FA and a new SRD in the OCT images at 24 months after the half-time PDT. No systemic or ocular side effects were found to be related to the treatment.

OCT showed that the SRD was completely resolved in 20 eyes (90%) at 1 month and in only 2 eyes (9%) at 3 months after the treatment. At 6 months after the half-time PDT, the SRD was resolved in all eyes. After that, a SRD recurred in only 1 eye (5%) at 18 months after the treatment. Because the SRD was small, the patient did not undergo a second treatment, and it naturally disappeared at 1 month after the recurrence. In all patients, there was no SRD at 24 months after the half-time PDT.

The half-time PDT was tolerated by all patients without the development of RPE atrophy or development of a CNV associated with the verteporfin treatment [7, 9]. In all patients, the retinal sensitivity in the central 10 degrees improved significantly at 24 months compared to that at the baseline. Unexpectedly, the retinal sensitivity in the central 10 degrees improved additionally at 24 months compared to that at 12 months in 17 patients (77%). However, the central 10 degrees retinal sensitivity was poorer at 24 months than at 12 months in 5 patients (23%). At 12 months after the treatment, the retinal sensitivity in the central 10 degrees of in 21 patients (95%) was significantly improved, and only in 1 patient (5%) had worse sensitivity than that at the baseline.

**Figure 1:**
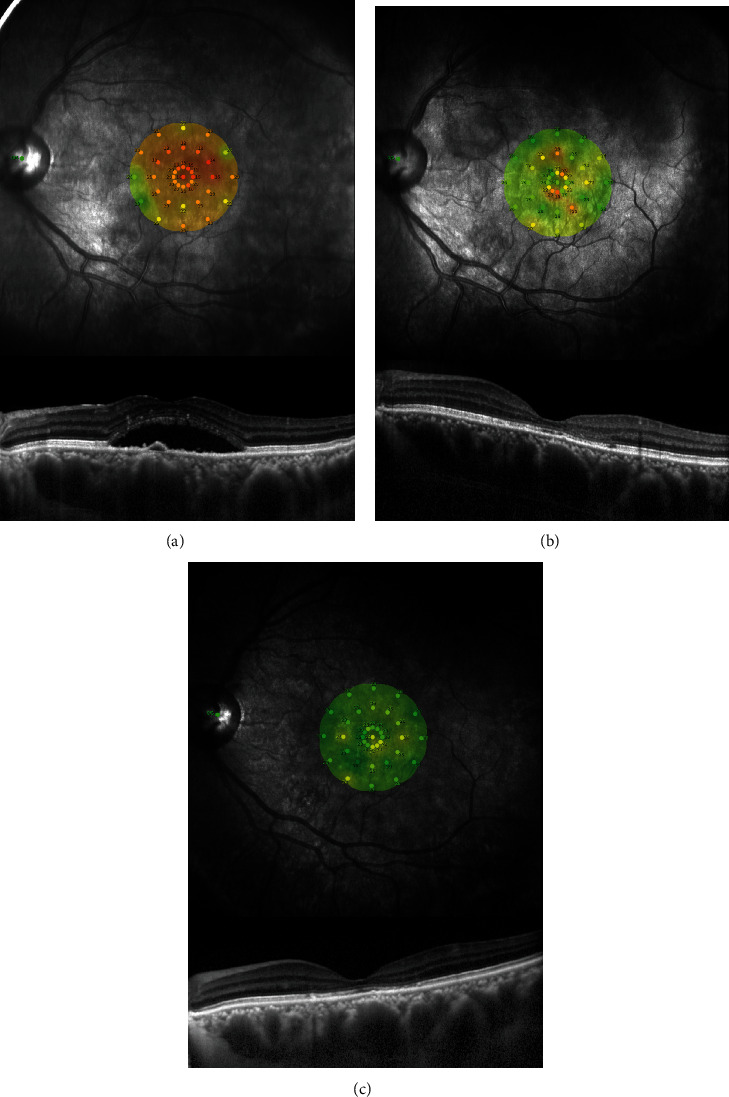
Findings in patients with chronic central serious chorioretinopathy (CSC) treated by half-time photodynamic therapy (PDT). Microperimetric images and optical coherence tomographic images of the left eye of a 43-year-old man with chronic CSC treated with half-time PDT at the baseline (a) and at 12 months (b) and 24 months (c) after treatment. His baseline decimal best-corrected visual acuity (BCVA) was 0.9, and the mean retinal sensitivity was 19.7 dB. After the half-time PDT, the symptoms were improved, and the serous retinal detachment was resolved. At 12 months after the treatment, the decimal BCVA was 1.2 and the retinal sensitivity was 26.3 dB. At 24 months, his BCVA was 1.2 and the retinal sensitivity was 27.6 dB.

**Figure 2:**
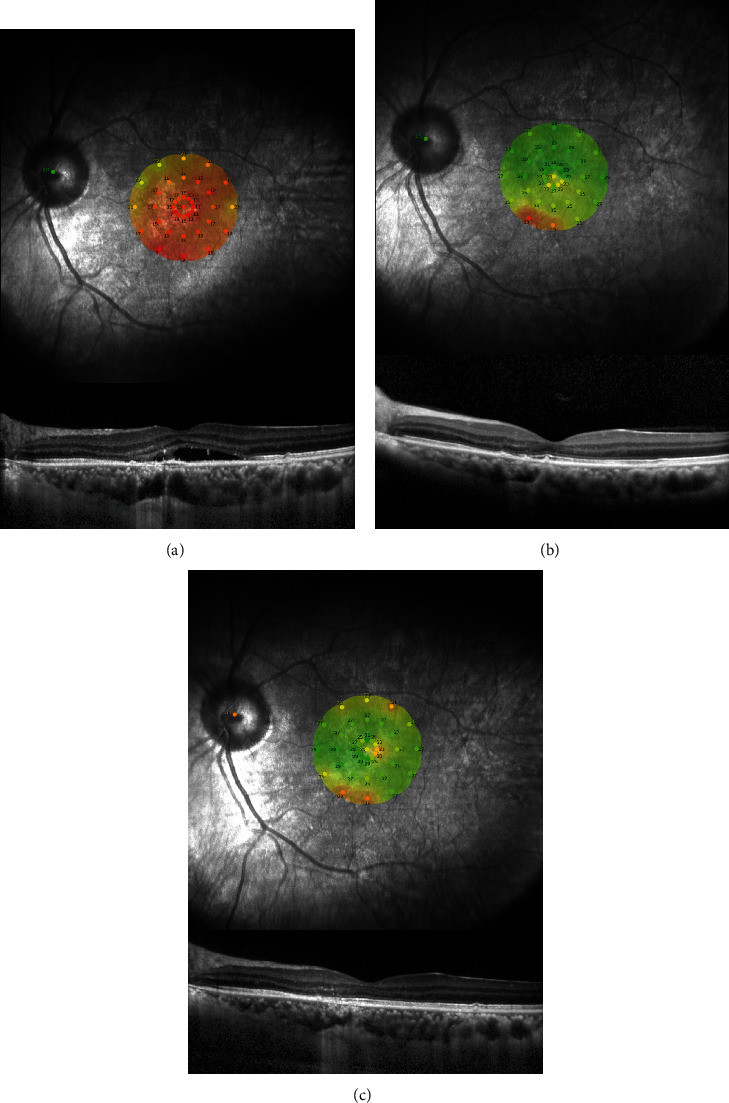
Findings in patients with chronic central serious chorioretinopathy (CSC) treated by half-time photodynamic therapy (PDT). Microperimetric images and optical coherence tomographic images of the left eye of a 52-year-old man with chronic CSC treated with half-time PDT at the baseline (a), at 12 months (b), and 24 months (c) after the treatment. His decimal best-corrected visual acuity (BCVA) was 0.6, and the retinal sensitivity was 15.5 dB at the baseline. After half-time PDT, the subjective symptoms were improved, and the serous retinal detachment was resolved. At 12 months after treatment, his decimal BCVA was 1.2 and retinal sensitivity was 26.6 dB. At 24 months, his decimal BCVA was 1.2 and the retinal sensitivity was 25.7 dB.

**Figure 3:**
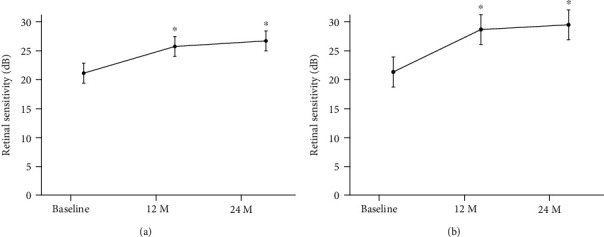
Means ± standard deviations of the retinal sensitivities before and after half-time photodynamic therapy (PDT) in eyes with chronic central serous chorioretinopathy (CSC). (a) Changes of mean retinal sensitivity in the central retinal area of 10 degrees before and after half-time PDT in patients with CSC. Bars are the standard deviations. (b) Changes of mean retinal sensitivity in the central retinal areas of 2 degrees before and after half-time PDT in patients with chronic CSC. Bars are standard deviations. There is a significant improvement in sensitivity compared with baseline after 12 and 24 months. ^∗^*P* < 0.001, M: months.

These changes in the retinal sensitivity during the follow-up period are shown in Figures 1 and 2, and the changes in the mean retinal sensitivity in the central 2 and 10 degrees during the follow-up period are shown in Table 2. The mean retinal sensitivities at the baseline was 19.28 ± 3.93 dB (median; 19.34 dB) in the central 2 degrees and 20.97 ± 2.92 dB (median; 20.9 dB) in the central 10 degrees. Both sensitivities were significantly improved at 12 months to 25.62 ± 2.79 dB (median; 26.11 dB) for the central 2 degrees and to 25.69 ± 2.25 dB (median; 25.65 dB) for the central 10 degrees (*P* < 0.001) and to 26.32 ± 2.62 dB (median; 27.38 dB) for 2 degrees and 26.66 ± 2.21 dB (median; 27.45 dB) for 10 degrees at 24 months (*P* < 0.001) after the half-time PDT (Figures 3(a) and 3(b)).

As described, the mean retinal sensitivities in the central 10 degrees were also significantly improved to 25.69 ± 2.25 dB (median; 25.65 dB) at 12 months and to 26.66 ± 2.21 dB (median; 27.45 dB) at 24 months (*P* < 0.01). The mean retinal sensitivity in the central 2 degrees was 25.62 ± 2.79 dB (median; 26.11 dB) at 12 months which was not significantly different from 26.32 ± 2.62 dB (median; 27.38 dB) at 24 months (*P* = 0.13).

**Figure 4:**
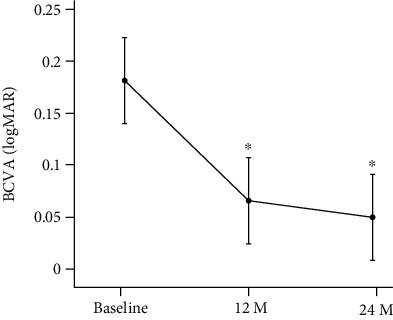
Changes of mean best-corrected visual acuity (BCVA) in logarithm of the minimum angle of resolution (logMAR) units before and after half-time photodynamic therapy (PDT) in eyes with chronic central serous chorioretinopathy (CSC). Bars are the standard deviations. There is a significant improvement in BCVA compared with baseline after 12 and 24 months. ^∗^*P* < 0.001, M: months.

The mean BCVA at the baseline was 0.18 ± 0.19 (median; 0.15) logMAR units, and it significantly improved to 0.065 ± 0.14 (median; 0) logMAR units at 12 months (*P* < 0.001) and to 0.049 ± 0.15 (median; -0.039) logMAR units at 24 months (*P* < 0.001) after the half-time PDT. The BCVA at 12 months was not significantly different from that at 24 months (*P* = 0.21) after the half-time PDT (Figure 4). The BCVA improved in 13 patients (59%), had not changed significantly in 8 patients (36%), and worsened in 1 patient (5%) at 12 months after the treatment. In 4 patients (18%), the BCVA improved at 24 months compared to that at 12 months and the BCVA worsened in 2 patients (9%) at 24 months compared to that at 12 months. In 16 patients (73%), the BCVA did not change at 24 months compared to that at 12 months. In 1 patient (5%), the BCVA worsened at 24 months compared to that at the baseline. In 15 patients (68%), the BCVA improved significantly at 24 months compared to the baseline. In 6 patients (27%), the BCVA did not change significantly at 24 months compared to baseline.

**Figure 5:**
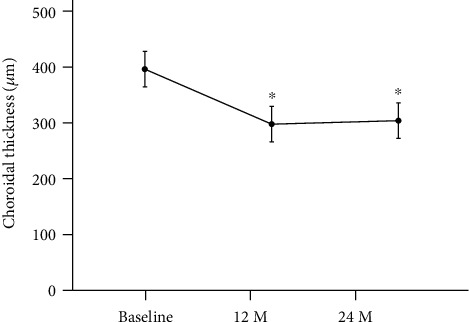
Changes of the subfoveal choroidal thickness (CT) before and after half-time photodynamic therapy (PDT) in eyes with chronic central serous chorioretinopathy (CSC). Bars are standard deviations. ^∗^*P* < 0.001, M: months. There is a significant decrease in the CT compared with baseline after 12 and 24 months.

The mean choroidal thickness (CT) at the baseline was 400.9 ± 92.2 *μ*m (median; 409 *μ*m), and it decreased significantly to 299.5 ± 98.0 *μ*m (median; 297 *μ*m) at 12 months (*P* < 0.001) and 305.8 ± 109.3 *μ*m (median; 288.5 *μ*m) at 24 months (*P* < 0.001) after the half-time PDT (Figure 5). The CT had decreased in 20 patients (91%) and had not decreased in 2 patients (9%) at 12 months and 24 months after the treatment.

The mean retinal thickness at 12 months and 24 months after the half-time PDT were 167.8 ± 33.1 *μ*m (median; 174 *μ*m) and 166.5 ± 37.6 *μ*m (median; 169 *μ*m).

There was no significant correlation between the retinal thickness and retinal sensitivity after the half-time reduced-fluence therapy. However, as reported, we had divided our patients into a continuous group, a fragmented group, and an absent group based of an earlier study on the integrity of the ellipsoid zone (EZ) at 12 months posttreatment [25]. There were 13 of our cases in the continuous group, 8 cases in the fragmented group, and 1 case in the absent group. The retinal sensitivity was significantly higher in the continuous group than the other two groups. (*P* = 0.01 Mann-Whitney *U* test).

The correlations between the BCVA and the retinal sensitivity in the central 2 and 10 degrees were not significant at the baseline (*P* = 0.30, *P* = 0.15; Table 3). On the other hand, there were significant negative correlations between the BCVA and the retinal sensitivity of the central 2 and 10 degrees at 12 months (*r* = −0.623, *P* < 0.01 and *r* = −0.574, *P* < 0.01; Table 3) and at 24 months (*r* = −0.620, *P* < 0.01 and *r* = −0.574, *P* < 0.01; Table 3). The changes in the BCVA from the baseline was not significantly correlated with the changes in retinal sensitivity in central 2 and 10 degrees at 12 months (*P* = 0.15, *P* = 0.07; Table 4) and at 24 months (*P* = 0.18, *P* = 0.11; Table 4).

## 4. Discussion

Our results showed that the mean retinal sensitivity in the central 2 and 10 degrees in eyes with chronic CSC was significantly better after half-time PDT at both 12 and 24 months than that at the baseline. The mean BCVA was also significantly better at 12 and 24 months than that at the baseline. These findings indicated that half-time PDT is effective in improving BCVA and retinal sensitivity in the macular region for at least 24 months. The CT was significantly thinner at 12 and 24 months than at the baseline. These results showed that the effect of this treatment on CT lasted for at least 24 months. Earlier studies had shown a decrease of the choroidal thickness after 1 week of PDT for CSC [26]. Unfortunately, we do not have any data of the retinal sensitivity at 1 week, so we cannot discuss about the thickness of the choroid and improved retinal sensitivity at an earlier period after PDT.

But, in this study, we mainly focused on the long-term course of PDT treatment for CSC, and even at 12 and 24 months after the PDT, the choroid was still thinner than that at the baseline. Thus, we believe that the effects of PDT had lasted not only in the long term, but also in the short term.

In all patients, the retinal sensitivity had improved at 24 months compared to that at baseline, but in 5 patients (23%), the retinal sensitivity was reduced at 24 months compared to retinal sensitivity at 12 months. In one of the 5 patients, the retinal sensitivity improved at 12 months and then decreased at 24 months. In 4 of 5 patients, the retinal sensitivity decreased once at 12 months and then increased at 24 months. The reason for these findings may be because there was a recurrence of the CSC which happened between the 6 and 12 months examination periods.

In the central 10 degrees, the mean retinal sensitivity was also significantly better at 24 months than at 12 months even though the BCVA at 24 months was not significantly different than the BCVA at 12 months. Even when the visual acuity reached a plateau at 12 months, some functions of the retina such as the retinal sensitivity can still improve until 24 months.

There have been many studies on the BCVA after half-time PDT treatments for chronic CSC with a posttreatment of more than two years [15, 27–33]. Although there were several studies that followed the retinal sensitivity for a long period [12, 14, 16, 23, 34–38], most of the follow-up period was at most one year. On the other hand, our experimental period extended for 24 months which provided information on the long-term effects of half-time PDT.

The different methods of using PDT for CSC include conventional, half-dose [12, 23], half-energy [14, 24], and half-time PDT [15, 16]. Previous reports showed that both these half-fluence PDT and conventional PDT were effective but half-fluence PDT had fewer side effects than conventional PDT. Senturk et al. and Fujita et al. evaluated the BCVA and retinal sensitivity before and after half-dose PDT for CSC at 6 and 12 months after the treatment [12, 23]. Reibaldi et al. and Hagen et al. reported that the BCVA and retinal sensitivity were improved at 12 months after half-fluence (half-energy) PDT [13, 37]. Sheptulin et al. reported that half-time PDT was effective in significantly improving the BCVA after 12 months [15]. Muslubas et al. also reported the improvement in retinal sensitivity 3 months after half-time PDT [16]. In these studies, the short-term and intermediate-term effects of half-dose PDT, half-fluence PDT, and half-time PDT were determined on the retinal sensitivity in eyes with CSC. To the best of our knowledge, our retinal sensitivity findings at 24 months are the first long-term findings.

The mean diameter of the laser spot in our study was 4148 ± 984 *μ*m while that of Senturk et al. was 2500 ± 455 *μ*m, Fujita et al. was 4371.4 ± 910.1 *μ*m, and Hagen et al. was 1700 ± 455 *μ*m. The exact calculation of the spot size reduced the risk of collateral damage such as choroidal ischemia, retinal atrophy, or development of choroidal neovascular [39]. Our spot size was larger than that of the other studies but the retinal sensitivity significantly improved at 1 month after the treatment although the retinal sensitivity in 3 patients (13.6%) worsened, 16 patients (72.7%) had an improvement, and the sensitivity of 3 patients was not recorded. At 12 months, the retinal sensitivity in the central 10 degrees significantly improved in 21 patients (95%). In all patients, the retinal sensitivity in the central 10 degrees improved at 24 months compared to that at the baseline. Eventually, the retinal sensitivity improved and there were no complications. Our average spot size was larger than that of earlier studies but there were no complications and the effect was maintained for a relatively long time.

In some patients with relatively good vision before treatment, the retinal sensitivity improved slowly and gradually for 24 months after the treatment. The reason for this is that the visual acuity reflects only the function of the central fovea, and the retinal sensitivity represents the status of a larger area of the foveal area. Thus, it is important to measure the retinal sensitivity in patients with chronic CSC and to evaluate the visual function in greater detail because the visual acuity does not necessarily reflect the overall visual function.

Many studies have examined the relationship between the BCVA and macular retinal sensitivity. Chung et al. and Kim et al. reported that the BCVA was significantly correlated with the mean retinal sensitivity around the macula in eyes with a resolved CSC [40, 41]. Chiba et al. showed that the BCVA was significantly correlated with the foveal sensitivity in macular thickening disorders [42]. Sugiura et al. also reported similar results between the BCVA and macular retinal sensitivity, but this significant relationship was not observed when the retinal sensitivity was calculated from a wider retinal area in eyes with chronic CSC [43].

There was a significant correlation between the BCVA and retinal sensitivity in our patients at 12 and 24 months, but they were not significantly correlated at the baseline. The reason why this lack of correlation is at the baseline might be because of the presence of the SRD. The changes in the BCVA were not significantly correlated with the changes in retinal sensitivity before and after the half-time PDT (Table 4). The reason for this discrepancy might be that there were many cases in which the visual acuity did not change significantly because they already had good visual acuity before treatment. Thus, we believe that it might be a ceiling effect. On the other hand, even at 24 months, the retinal sensitivity had increased more than at 12 months. From these results, we conclude that half-time PDT is a very useful method to treat patients with chronic CSC even those with relatively good visual acuity.

There was no significant difference between the retinal sensitivity at 2 and 10 degrees before the treatment but there was a tendency that retinal sensitivity at 2 degrees was lower than the retinal sensitivity at 10 degrees. (*P* = 0.057 Mann-Whitney *U* test). The reason why there was this difference might be the effects of the SRD. It has been recently reported that there was a significant correlation between the retinal sensitivity and the SRD height [26]. On the other hand, there was no significant difference between retinal sensitivity at 2 and 10 degrees at 12 months after the treatment because of the absence of the SRD (*P* = 0.98; Mann-Whitney *U* test).

There was no significant correlation between the retinal thickness and retinal sensitivity after the half-time reduced-fluence therapy. However, as reported, we had divided our patients into a continuous group, a fragmented group, and an absent group based on an earlier study on the integrity of the ellipsoid zone (EZ) at 12 months posttreatment [25]. There were 13 of our cases in the continuous group, 8 cases in the fragmented group, and 1 case in the absent group. The retinal sensitivity was significantly higher in the continuous group than the other two groups (*P* = 0.01 Mann-Whitney *U* test). The improvements of the retinal sensitivity were associated with an improvement in the integrity of the EZ [40, 44]. We were not able to detect the IZ consistently in all our patients posttreatment, and thus cannot determine if there was a change in the IZ posttreatment. Before the treatment, we could not see either the EZ or IZ because the retina was completely detached at the macular area by the existence of SRD.

As we mentioned, there was no specific relationship between the retinal structure and retinal sensitivity before treatment. We believe that the lack of correlation between visual acuity and retinal sensitivity means that the SRD has only a slight effect on the BCVA. We believe that the retinal sensitivity might be a method that can detect changes in the visual function that cannot be detected by the BCVA.

Our study has several limitations. First, the sample size was small, and this was a retrospective study without a control group. The lack of a control group may limit generalizations and definitive comparisons of half-time PDT with other treatment methods. Further prospective studies with a control group or other modified treatment groups, such as half-dose or half-fluence PDT in a larger sample, will be needed to determine the efficacy of half-time PDT on the retinal sensitivity in eyes with CSC. A second limitation was that the significant increase in the mean central retinal sensitivity over 24 months in eyes with chronic CSC might be a learning effect or test-retest variations [45, 46]. This can be best examined by having a control group but then ethical problems will arise.

In conclusion, the retinal sensitivity and BCVA improved and were maintained for at least 24 months after half-time PDT in eyes with chronic CSC. Our findings indicate that it is important to examine the retinal sensitivity when evaluating the effect of half-time PDT in eyes with chronic CSC. Although further evaluations with a larger sample size are needed, our results showed that half-time PDT was beneficial for resolving chronic CSC for at least 24 months.

## Figures and Tables

**Table 1:** Demographics of the patients with central serous chorioretinopathy.

Number of eyes	22
Mean ± SD age (years)	57.5 ± 11.0
Sex	Men 18; women 4
Mean ± SD spot size diameter (*μ*m)	4148 ± 984 (median; 4250)
Mean ± SD baseline BCVA (logMAR)	0.18 ± 0.19 (median; 0.15)
Mean ± SD baseline retinal sensitivity 2 degrees (dB)	19.28 ± 3.93 (median; 19.34)
Mean ± SD baseline retinal sensitivity 10 degrees (dB)	20.97 ± 2.92 (median; 20.9)
Mean ± SD baseline choroidal thickness (*μ*m)	400.9 ± 92.1 (median; 409)

SD: standard deviation; BCVA: best-corrected visual acuity; logMAR: logarithm of the minimum angle of resolution.

**Table 2:** Mean retinal sensitivity and visual acuity in patients with central serous chorioretinopathy before and after half-time photodynamic therapy.

	Baseline	12 months	24 months
Mean ± SD retinal sensitivity 2 degrees (dB)	19.28 ± 3.93 (median; 19.34)	25.62 ± 2.79 (median; 26.11)	26.32 ± 2.62 (median; 27.38)
Mean ± SD retinal sensitivity 10 degrees (dB)	20.97 ± 2.92 (median; 20.9)	25.69 ± 2.25 (median; 25.65)	26.66 ± 2.17 (median; 27.45)
Mean ± SD BCVA (logMAR)	0.18 ± 0.19 (median; 0.15)	0.065 ± 0.14 (median; 0)	0.049 ± 0.15 (median; -0.039)
Mean ± SD baseline choroidal thickness (*μ*m)	400.9 ± 92.1 (median; 409)	299.5 ± 98.0 (median; 297)	305.8 ± 109.3 (median; 288.5)

SD: standard deviation; BCVA: best-corrected visual acuity; logMAR: logarithm of the minimum angle of resolution.

**Table 3:** Correlation between best-corrected visual acuity and retinal sensitivity.

	Baseline	12 months	24 months
2 degrees	-0.226 (*P* = 0.3)	-0.623 (*P* < 0.01)	-0.62 (*P* < 0.01)
10 degrees	-0.313 (*P* = 0.15)	-0.574 (*P* < 0.01)	-0.568 (*P* < 0.01)

BCVA: best-corrected visual acuity; logMAR: logarithm of the minimum angle of resolution.

**Table 4:** Correlation between the changes in BCVA and retinal sensitivity.

	From 12 months to baseline	From 24 months to baseline
2 degrees	-0.313 (*P* = 0.15)	-0.29 (*P* = 0.18)
10 degrees	-0.389 (*P* = 0.07)	-0.347 (*P* = 0.11)

BCVA: best-corrected visual acuity; logMAR: logarithm of the minimum angle of resolution.

## Data Availability

The datasets generated during and/or analyzed during the current study are available from the corresponding author on reasonable request.
